# Gene expression silencing therapy in tumors, focus on gastrointestinal and genitourinary tumors

**DOI:** 10.3389/fimmu.2025.1657040

**Published:** 2025-10-03

**Authors:** Nathan El-Ghazzi, Antoine Italiano, Eurydice Angeli

**Affiliations:** ^1^ Early Phase Trials Department, Institut Bergonié, Bordeaux, France; ^2^ Department of Medical Oncology, Institut Bergonié, Bordeaux, France; ^3^ Université Sorbonne Paris Nord, UFR UFR Santé Médecine et Biologie Humaine (SMBH), Bobigny, France; ^4^ Medical Oncology Department, Hôpital Avicenne (APP-HP), Bobigny, France

**Keywords:** gene silencing therapy, antisense oligonucleotide, siRNA, miRNA, gastrointestinal cancer, genitourinary cancer

## Abstract

Precision oncology has seen significant progress with oligonucleotide-based therapies, which provide a novel approach to gene expression silencing. These therapies, including antisense oligonucleotides (ASOs), small interfering RNAs (siRNAs), and microRNAs (miRNAs), target specific genetic sequences with high precision. They offer promising solutions for cancers resistant to conventional treatments due to their ability to modulate previously “undruggable” targets and their reduced toxicity. However, challenges such as susceptibility to degradation, poor cellular uptake, and off-target effects have hindered their clinical application. Advances in chemical modifications and delivery systems, like lipid nanoparticles and GalNAc conjugates, have improved the stability and efficacy of these therapies. This review discusses the structural features, mechanisms of action, and clinical applications of ASOs, siRNAs, and miRNAs, focusing on gastrointestinal and genitourinary cancers. We highlight successful oncology applications, such as siRNA-based therapies targeting specific oncogenes, which have shown promise in clinical trials. Continued advancements in this field are paving the way for more effective and safer cancer treatments.

## 1 Introduction

Recent advances in molecular medicine have significantly contributed to the rise of precision oncology. Cancer is characterized by the accumulation of molecular alterations that confer selective advantages to tumor cells, enabling them to evade conventional therapies. Consequently, the field of precision medicine has rapidly expanded: among the 198 newly FDA-approved drugs between 1998 and 2022, 43% were classified as targeted precision therapies (1). Gene expression silencing represents a promising new therapeutic avenue in precision oncology. This approach leverages oligonucleotide-based drugs—short single-stranded DNA or RNA molecules capable of binding to specific DNA, RNA, to modulate gene expression. These therapies fall mainly into three main categories (2): (i) antisense oligonucleotides (ASOs), (ii) small interfering RNAs (siRNAs), and (iii) microRNAs (miRNAs). Their main advantages include high target specificity, reduced toxicity, and the ability to modulate previously “undruggable” targets, even those refractory to conventional targeted therapies. Despite their relatively simple design and ease of synthesis, oligonucleotide-based therapeutics have faced multiple challenges in clinical translation. Substantial progress has been achieved through successive generations of chemical modifications aimed at enhancing potency and minimizing toxicity. This review will first describe the different gene expression silencing molecules developed to date, their structural features, mechanisms of action, and limitations of these three therapeutic classes. We will then highlight their current clinical applications, with a specific focus on gastrointestinal (GI) and genitourinary (GU) cancers.

## 2 Different gene expression silencing molecules available, brief description

### 2.1 Antisense oligonucleotides

**Figure 1:**
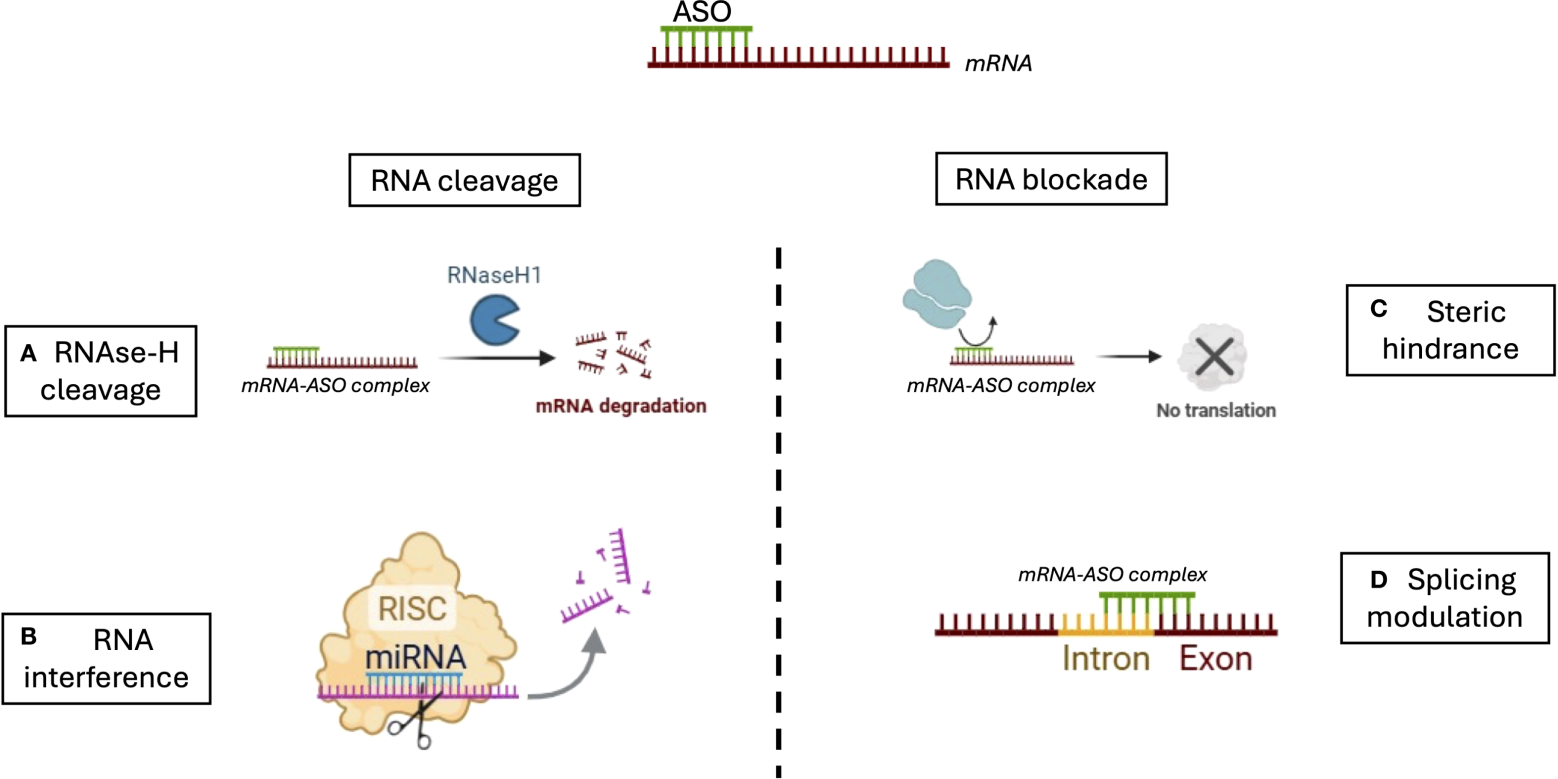
Mechanisms of action of antisense oligonucleotides (ASOS). ASOs regulate gene expression through two main strategies: RNA cleavage and RNA blockade. **(A)** In the RNase H-dependent pathway, ASOs hybridize with target mRNA to form a DNA-RNA duplex, which is recognized and cleaved by RNase H1, leading to mRNA degradation. **(B)** In RNA interference (RNAi), siRNAs or miRNAs guide the RNA-induced silencing complex (RISC) to the target mRNA, promoting its cleavage or translational repression. **(C)** ASOs can act through steric hindrance by binding to critical regions of the mRNA, such as the translation initiation site or regulatory motifs, thereby physically blocking the 40S ribosomal subunit or splicing factors (e.g., snRNPs, SR proteins, hnRNPs) from accessing their binding sites. This prevents translation or alters splicing without degrading the transcript. **(D)** ASOs can also modulate pre-mRNA splicing by targeting splice donor or acceptor sites, or splicing enhancers/silencers, resulting in exon skipping, intron retention, or inclusion of alternative exons, ultimately modifying protein isoforms or restoring proper splicing in genetic diseases.

Antisense oligonucleotides (ASOs) have been under development for over two decades. The first major therapeutic success was nusinersen, approved for the treatment of spinal muscular atrophy (SMA), an autosomal recessive genetic disorder caused by insufficient levels of survival motor neuron (SMN) protein. Nusinersen is an ASO that modulates the alternative splicing of the *SMN2* pre-mRNA, thereby enhancing the production of functional, full-length SMN protein (3, 4). This therapeutic approach, when administered as early as possible in affected newborns, has shown a significant improvement in prognosis. It has led to the implementation of genetic testing as part of a nationwide newborn screening program in the United States since 2018. The fundamental principle of ASO therapy is relatively straightforward (**Figure 1**): ASOs are short, single-stranded DNA oligonucleotides, typically 13–25 nucleotides in length, designed to hybridize with complementary sequences of target messenger RNA (mRNA) (5). Once bound, ASOs can modulate gene expression through different mechanisms. The most common approach relies on RNase H–mediated degradation. RNase H is a ubiquitous endonuclease that selectively cleaves the RNA strand of RNA/DNA heteroduplexes. ASOs that exploit this mechanism are known as gapmers. These typically consist of a central region of unmodified DNA flanked by chemically modified RNA-like nucleotides on both ends, which enhance stability and binding affinity. Upon hybridization to the target mRNA, the RNA/DNA duplex recruits RNase H, resulting in RNA cleavage, degradation, and subsequent suppression of gene expression (6, 7). ASOs can also function through RNase H–independent mechanisms, most notably by modulating pre-mRNA splicing. During gene expression, pre-mRNAs are processed in the nucleus to generate mature mRNA. Alternative splicing allows a single gene to produce multiple protein isoforms. Splice-switching ASOs can redirect this process by binding to specific splicing motifs, thereby blocking access of splicing factors and altering exon inclusion or exclusion. This strategy has shown therapeutic potential in diseases where splicing defects are critical drivers (8). Finally, ASOs may act indirectly by interfering with the RNA-induced silencing complex (RISC). RISC regulates gene expression post-transcriptionally by guiding miRNA-mediated mRNA degradation or translational repression. By binding to and displacing endogenous miRNAs from RISC, ASOs can inhibit pathological miRNA activity and restore expression of their target genes (9). This mechanism is particularly relevant in cancer, where oncogenic miRNAs contribute to aberrant gene repression.

### 2.2 Small interfering RNA

**Figure 2:**
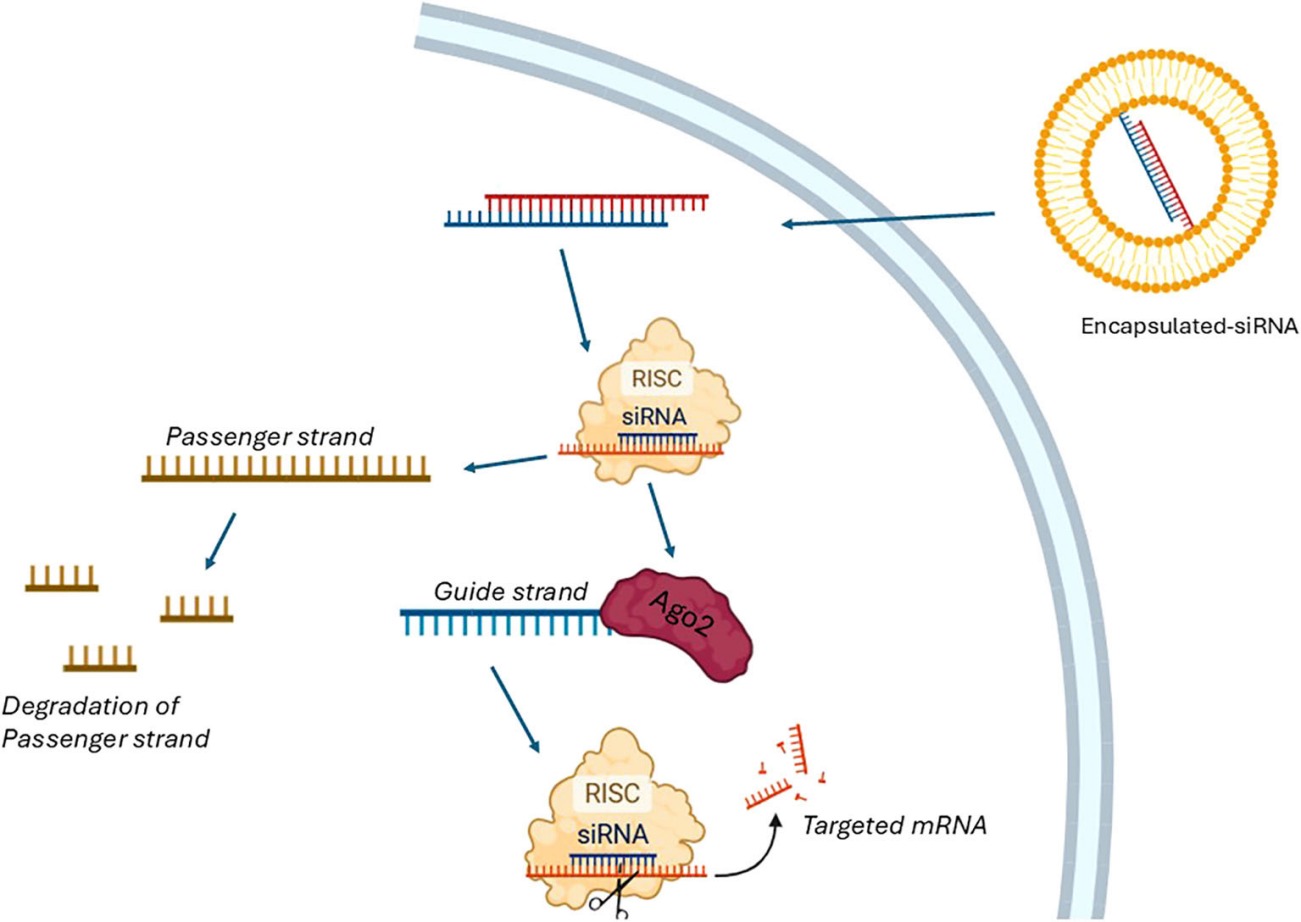
Process of siRNA Therapy. The process of siRNA therapy begins with the introduction of encapsulated double-stranded siRNA into the cellular environment. Once inside the cell, the siRNA duplex is incorporated into the RNA-induced silencing complex (RISC). With the assistance of a helicase and utilizing the energy from ATP hydrolysis, the siRNA duplex is unwound into two single strands: the passenger strand and the guide strand. The passenger strand is subsequently degraded, while the guide strand remains associated with the Argonaute 2 (Ago2) protein, a key component of RISC. The guide strand of the siRNA directs RISC to the complementary targeted mRNA sequence, leading to its cleavage and degradation. This process effectively silences gene expression by preventing the translation of the targeted mRNA.

Building on the concept of direct mRNA targeting by ASO, siRNAs employ a cellular machinery-based approach to degrade specific transcripts, offering an alternative mechanism for gene silencing. siRNAs are short double-stranded RNA molecules, typically 19–39 nucleotides in length, that mediate RNA interference (RNAi) and gene silencing (10). Compared with small-molecule drugs or monoclonal antibodies, siRNAs have the intrinsic advantage of acting through perfect base pairing with target mRNA, whereas conventional drugs must recognize specific three-dimensional protein conformations, which are not always present in pathological settings. In theory, any gene can be selectively silenced by a rationally designed siRNA. Each siRNA is composed of two strands: a sense (passenger) strand and an antisense (guide) strand (**Figure 2**). Once in the cytoplasm, the siRNA duplex is incorporated into the RISC, where the two strands are separated. The passenger strand is degraded, while the antisense strand is retained and loaded onto Argonaute 2 (AGO2). Guided by base complementarity, AGO2 directs RISC to the target mRNA and cleaves it, thereby preventing translation and downregulating gene expression (11, 12). As with ASOs, siRNAs can be chemically modified to improve *in vivo* stability and reduce renal clearance. However, their delivery poses greater challenges due to their relatively high molecular weight (~13 kDa), strong negative charge, and dimensions (~7–8 nm in length, 2–3 nm in diameter), which limit passive diffusion across cell membranes. In addition, naked siRNAs are rapidly eliminated by renal clearance, as the glomerular filtration barrier efficiently removes molecules below 8 nm. To overcome these barriers, siRNA therapeutics require specialized drug delivery systems. Lipid-based nanoparticles, dynamic polyconjugates, and exosome vesicles are among the most effective and safe approaches for facilitating intracellular delivery of siRNAs (10, 13).

### 2.3 microRNAs

While siRNAs are exogenous molecules designed for precise gene knockdown, microRNAs (miRNAs) are small non-coding RNAs that regulate gene expression at the post-transcriptional level, modulating multiple targets simultaneously and reflecting a more nuanced layer of endogenous control. They are typically 18 to 24 nucleotides in length and can bind to target sequences located in the 3′ untranslated region (3′UTR), 5′UTR, or the open reading frame of messenger RNAs (14). It is estimated that approximately 30% of human mRNAs are under physiological regulation by endogenous miRNAs. These molecules are involved in a wide array of cellular processes, including differentiation, apoptosis, proliferation, and even DNA repair (15). In cancer, miRNAs can act as either tumor suppressors or oncogenes. Tumor-suppressive miRNAs downregulate oncogene expression, while oncogenic miRNAs repress tumor suppressor genes, thereby promoting tumor progression. Although siRNAs generally achieve stronger and more specific silencing, miRNAs exert broader regulatory effects because of their partial complementarity with target transcripts. A single miRNA can simultaneously regulate multiple mRNAs, leading to translational repression or mRNA destabilization and reshaping entire gene networks (16). Two main therapeutic strategies have been developed based on miRNA function: (i) replacement therapy, which involves reintroducing tumor suppressor miRNAs that are downregulated in cancer cells, and (ii) inhibition of oncogenic miRNAs to suppress tumor growth (17–19). The short length of miRNAs contributes to their relative stability and resistance to ribonuclease-mediated degradation. Beyond therapeutic applications, miRNAs are also being explored as diagnostic and prognostic biomarkers, with potential utility in predicting survival, monitoring drug resistance, and facilitating early cancer detection (20). Their limited specificity resulting from simultaneous activity on multiple pathways and the need for protection against rapid clearance in the bloodstream currently make miRNAs the least clinically advanced modality among gene-silencing approaches.

Together, ASOs, siRNAs, and miRNAs illustrate the diverse strategies available to therapeutically modulate gene expression, each with unique advantages, limitations, and potential clinical applications. (**Figures 1**, **2**, **Table 1**).

**Table 1:** Advantages and disadvantages of ASOs, siRNAs, and miRNAs.

Therapy Type	Advantages (+)	Disadvantages (-)
Antisense Oligonucleotides (ASOs)	+ Precise targeting of specific mRNAs; modulates splicing and RNA degradation (RNase H–dependent) + Chemically modifiable for improved stability (e.g., phosphorothioate, 2′-O modifications) + Clinically validated (e.g., nusinersen for SMA)+ Can restore gene expression by blocking oncogenic miRNAs	- Delivery challenges, especially outside liver tissues - Risk of immune activation and thrombocytopenia - Off-target effects due to binding unintended RNA or proteins
Small Interfering RNA (siRNA)	+ Highly potent and specific gene silencing via RISC-mediated mRNA cleavage + Synthetic and customizable + Chemical modifications and lipid nanoparticle delivery enhance stability and uptake	- Poor membrane permeability and rapid renal clearance without delivery systems - Complex intracellular delivery needed - Possible immune stimulation and off-target effects
microRNA (miRNA)	+ Can regulate multiple genes simultaneously due to partial complementarity + Endogenous molecules with lower immunogenicity + Potential for replacement or inhibition strategies +Emerging biomarker applications	- Lower specificity may cause off-target gene regulation - Requires protection against degradation in bloodstream -Least clinically developed among oligonucleotide therapies

## 3 General challenges, engineering strategies, and safety considerations

The intrinsic properties of single-stranded DNA and RNA oligonucleotides complicate their use as therapeutic agents. These synthetic nucleic acids are negatively charged molecules with physicochemical characteristics that differ substantially from small-molecule drugs or conventional chemotherapies. In addition, oligonucleotides must cross the cell membrane to reach their intracellular targets, which presents another major barrier. Early limitations included rapid nuclease-mediated degradation, poor cellular uptake, unfavorable biodistribution, and suboptimal binding affinities to complementary sequences (6, 21). To overcome these limitations, several chemical engineering strategies have been developed.

### 3.1 Limiting nucleases degradation

#### 3.1.1 Phosphorothioate modifications

Substitution of a non-bridging oxygen atom in the phosphate backbone with sulfur generates phosphorothioate (PS) linkages, one of the most widely used modifications in oligonucleotide design. PS linkages increase resistance to nuclease degradation (extending half-lives from minutes to days) and enhance plasma protein binding, which prolongs circulation time and reduces hepatic clearance, thereby improving tissue uptake (22–24).

#### 3.1.2 Sugar modifications

Ribose modifications at the 2′-hydroxyl group are commonly used to enhance nuclease resistance. The most frequent variants—2′-O-methyl and 2′-O-methoxyethyl—also increase duplex thermal stability and prevent RNase H-mediated degradation. These modifications are particularly useful for “steric-blocking” strategies, such as modulation of alternative splicing (2, 24, 25). Another approach involves bridging the 2′-oxygen and 4′-carbon atoms to generate bridged nucleic acids (BNAs). Notable examples include locked nucleic acids (LNAs, with 2′,4′-methylene linkages) and 2′,4′-constrained ethyl nucleic acids (S-cETs). These structures promote favorable binding conformations, improve hybridization efficiency, and allow the design of shorter oligonucleotides (as short as 13-mers) (24, 26, 27).

### 3.2 Facilitating cancer cell delivery

Efficient intracellular delivery remains one of the greatest hurdles in oligonucleotide therapeutics. Several novel delivery platforms have emerged, including exosomes and viral vectors (28, 29) but the most consistent clinical results have been achieved with N-acetylgalactosamine (GalNAc) conjugation and lipid nanoparticles (LNPs). GalNAc-conjugated oligonucleotides exploit the asialoglycoprotein receptor (ASGPR), which is highly expressed on hepatocytes and mediates internalization of GalNAc-containing ligands. Covalent attachment of GalNAc moieties to oligonucleotides has significantly improved *in vivo* distribution (30, 31). Importantly, no GalNAc-mediated off-target effects have been reported, even in activated T cells, despite their known ASGPR expression (32). While data on ASGPR expression in tumor cells remain limited, *in vitro* studies have shown that HepG2 (hepatocellular carcinoma), MCF-7 (breast cancer), and A549 (lung cancer) cells can internalize GalNAc-conjugated probes—including fluorescent markers, ASOs, and siRNAs—in an ASGPR-dependent manner, with uptake proportional to receptor expression (33). LNPs have also become a central delivery platform. These structures consist of amphipathic lipids with hydrophilic head groups and hydrophobic alkyl chains. Their cationic properties facilitate electrostatic interactions with negatively charged oligonucleotides, allowing encapsulation of siRNAs and other nucleic acids. LNPs protect oligonucleotides from nuclease degradation, enhance cellular uptake, and promote endosomal escape (24, 34, 35). The successful application of LNP technology in mRNA vaccines underscores its broad potential for future cancer therapies (36). Finally, viral vectors represent another promising modality. Recombinant adeno-associated viruses (AAVs), which remain episomal without genomic integration, combine features of oligonucleotide and gene therapies. A notable example is U7 small nuclear RNA, which has been engineered to induce exon skipping in preclinical models and is currently under clinical investigation for Duchenne muscular dystrophy (37–39). Even with efficient entry, intracellular barriers, such as RNase degradation and endosomal entrapment remain significant challenges (23, 40).

### 3.3 Safety concerns

Off-target effects remain a central safety concern in oligonucleotide therapy. These can arise through unintended binding to surface proteins—which naturally interact with nucleic acids—potentially activating innate immune pathways via Toll-like receptors (TLRs) (41). Additional mechanisms include hybridization to unintended RNA targets, leading to aberrant gene modulation, or competition with endogenous RNAs for cellular machinery, potentially interfering with physiological miRNA pathways. The latter, however, has not been clinically observed to date (42). Careful sequence optimization is therefore critical to minimize such risks (6). Thrombocytopenia has emerged as a notable class toxicity, particularly with ASOs. This phenomenon is thought to be related either to a direct interaction between the ASO and recognized platelet surface proteins, or indirectly through activation of Toll-like receptors (TLRs), mimicking consumption thrombocytopenia seen in inflammatory states (43). The telomerase inhibitor imetelstat, for example, was associated with a treatment-related fatal intracranial hemorrhage due to grade 4 thrombocytopenia in a phase II trial (44). Importantly, this toxicity appears specific to ASOs, as siRNA-based therapies have not demonstrated similar adverse hematologic profiles to date. this discrepancy may partly reflect the longer development history and wider clinical exposure of ASOs, especially those incorporating 2′-MOE chemistry (45). Interestingly, 2′-MOE modifications may also mitigate immune-related thrombocytopenia by reducing TLR9 activation and dampening innate immune responses.

## 4 Applications of gene expression silencing in GI/GU tumors

### 4.1 Genitourinary tumors 

#### 4.1.1 Prostate cancer

##### 4.1.1.1 ASO

The androgen receptor (AR) is a nuclear transcription factor whose deregulation plays a key role in prostate cancer. Its direct targeting with anti-androgens or indirect targeting through inhibition of its ligand (testosterone) is well established and widely used. However, acquired resistance mechanisms such as *AR* mutations and amplification often emerge. Targeting upstream of the protein therefore represents an interesting alternative strategy. To date, the most clinically advanced gene expression silencing molecule in castration resistant prostate cancer (CRPC) is apatorsen (OGX-427), targeting heat shock protein 27 (Hsp27). Hsp27 acts as a shuttle to transport activated AR into the nucleus—an essential step for its function as a transcription factor. Apatorsen is a 2′-O-methoxyethyl (2′-MOE) modified ASO that inhibits Hsp27 expression. In a phase I trial involving 42 patients with multitreated metastatic CRPC, receiving apatorsen, safety was acceptable, and 12 patients achieved stable disease. A PSA reduction of more than 50% was observed in 10% of CRPC patients (46). The drug was advanced into phase II PACIFIC trial for patients with metastatic CRPC and PSA-only progression under abiraterone. They were randomized to receive abiraterone alone or in combination with apatorsen. Progression-free survival at day 60 was 17% in the control arm versus 33% in the apatorsen arm. Despite being modest, such results in a heavily pretreated population, suggest biological activity, and warrant further investigation to identify predictive biomarkers (47). The same research group also identified DEAD-box helicase 5 (*DDX5*) as a relevant target in CRPC. *DDX5* overexpression is associated with disease progression through enhanced DNA damage repair mechanisms. A *DDX5*-specific ASO successfully reduced CRPC cell viability *in vitro* and led to tumor regression in patient-derived xenograft (PDX) models. These findings open avenues for combination strategies with other DNA damage response inhibitors or DNA-damaging agents, including radionuclide therapies (48). More recently, a Japanese team developed an ASO directly targeting mouse androgen receptor in genetically engineered CRPC models. This *AR*-specific ASO demonstrated efficacy against mutated AR variants in CRPC, both *in vitro* and in PDX models. Moreover, their work highlighted a feedback loop between *AR* signaling and the *PI3K/AKT* pathway, providing a rationale for combining *AR*-targeted ASOs with *AKT* inhibitors, which showed synergistic preclinical activity (49).

##### 4.1.1.2 siRNAs

Given the central role of *AR* in prostate cancer, it has also been targeted using siRNAs. A phase I clinical trial (NCT02866916) evaluated an *AR*-targeting siRNA called SXL01 (PROSTIRNA). However, the NCT02866916 trial was withdrawn in early development, likely due to strategic, financial, or operational reasons common to early-phase siRNA programs, though no specific safety concerns were publicly reported (50). Another therapeutic target, *EphA2*, is a tyrosine kinase receptor overexpressed in various cancers, including prostate cancer, and is involved in promoting proliferation, survival, and migration. Initial *in vitro* success led to a phase I clinical trial (NCT01591356) involving multiple tumor types, including prostate, melanoma, pancreatic, and bladder cancers. Results are pending at the time of writing (51). Several siRNA-based approaches are still in the preclinical phase:

- siRNA targeting of *PARP1*, a key enzyme involved in DNA damage repair and genome stability, has been shown to suppress proliferation and invasion in prostate cancer cells regardless of *BRCA* mutation status (52).
- Clusterin, a cytoprotective chaperone upregulated following AR inhibition, was silenced using a lipid nanoparticle-delivered siRNA, showing efficacy in CRPC models (53).
- *TRIM24* was silenced using a nanocarrier-based system conjugated to a PSMA-targeting human monoclonal antibody. Knockdown of *TRIM24* reduced cell proliferation, colony formation, and invasion in PSMA-positive CRPC cells and decreased tumor burden and bone loss in a CRPC bone metastasis model (54).

##### 4.1.1.3 miRNAs

More than 50 miRNAs have been identified as dysregulated in prostate cancer, including both oncogenic and tumor-suppressive miRNAs (55). Several miRNAs are involved in modulating AR signaling. For instance, miR-21 has been shown to regulate *AR* by downregulating TGF-β signaling, thereby reducing its growth-inhibitory effects and contributing to PC progression (56). As such, miRNA-based therapies aim either to inhibit oncogenic miRNAs or to restore tumor-suppressive miRNAs. The predominant approach is the use of anti-miRNA oligonucleotides designed to bind and neutralize oncogenic miRNAs. These agents, known as antagomirs, are chemically modified for increased stability and improved delivery (57, 58). For example, administration of anti-miR-221 and anti-miR-222 has demonstrated tumor growth inhibition in the PC3 prostate cancer cell line (59). Conversely, miRNA replacement strategies have also been explored. Delivery of miR-34 into prostate tumor-bearing mice resulted in reduced tumor growth and decreased bone metastasis (60). A novel delivery platform has been reported by an American team, which conjugated a chemically modified miR-34a to DUPA (2-[3-(1,3-dicarboxypropyl)ureido]pentanedioic acid). DUPA is a small-molecule ligand that binds specifically to PSMA (61). In combination with nigericin, DUPA-conjugated-miR-34a facilitates endosomal escape. This strategy improved therapeutic efficacy *in vitro* and *in vivo* (61). A recent innovation involves a fluoroquinolone derivative designed as a small molecule inhibitor of miR-21 (62). Despite the promising potential of miRNA therapies in CRPC, clinical translation remains limited without any phase I reported at the best of our knowledge.

#### 4.1.2 Bladder cancer

##### 4.1.2.1 ASO

As previously mentioned, apatorsen, an ASO targeting Hsp27, has also been investigated in localized non-muscle invasive bladder cancer (NMIBC) since urothelial cancer has been known to express Hsp27. In a phase I neoadjuvant study, intravesical apatorsen induced a pathological complete response in 5 of 13 treated patients (38%), highlighting the potential for a biomarker-guided, surgical-sparing approach in carefully selected responders (63). The Borealis-1 trial assessed apatorsen in combination with gemcitabine and cisplatin as first-line therapy for metastatic bladder cancer (64). Although no statistically significant survival benefit was observed, a favorable trend was noted in patients with poor prognostic features. More recently, the phase II Borealis-2 trial evaluated apatorsen combined with docetaxel versus docetaxel alone. The combination led to a 20% more reduction in the risk of death (HR 0.80; 95% CI 0.65–0.98), suggesting a clinical benefit (46, 65). Given that docetaxel is not a standard of care in bladder cancer, combinations with newly approved agents such as enfortumab-vedotin and checkpoint inhibitors may represent a promising therapeutic strategy (66).

Livin, a member of the inhibitor of apoptosis proteins (IAP) family, is minimally expressed in normal tissues but highly upregulated in bladder cancer. By inhibiting caspases and blocking apoptosis, Livin contributes to tumor cell survival, promotes resistance to chemotherapeutic agents, and has been associated with increased proliferation, invasion, and metastatic potential. Preclinical studies demonstrated that a Livin-targeting ASO successfully induced apoptosis *in vitro* and inhibited tumor growth *in vivo* in murine models expressing high levels of Livin by immunohistochemistry (67). These results support further development of Livin-directed ASO therapy.

##### 4.1.2.2 siRNA

A novel siRNA strategy entered phase I clinical trials for BCG-unresponsive non-muscle invasive bladder cancer (NCT06351904). The therapy, RAG-01, is a small activating RNA designed to restore p21 expression, a tumor suppressor gene. Preliminary results presented in 2025 showed complete responses in nearly two-thirds of patients in the lowest-dose cohorts, with an encouraging safety profile. A phase II expansion is underway.

Additional preclinical siRNA studies have targeted: *PLK-1*, a key mitotic kinase, and *Snail-1*, a transcription factor involved in epithelial-mesenchymal transition (EMT), both demonstrating efficacy in inhibiting tumor growth (68, 69).

##### 4.1.2.3 miRNA

To date, no miRNA-based therapy has reached clinical trials in bladder cancer. However, several preclinical studies show promising avenues: miR-145 acts as a tumor suppressor in non-muscle invasive bladder cancer by inhibiting progression. A Japanese team demonstrated that intravesical delivery of lipid nanoparticle-encapsulated miR-145 (LNP-miR-145) suppressed growth of premalignant bladder lesions in murine models without systemic toxicity (70). miR-424-5p is downregulated in cisplatin-resistant bladder cancer cells. Restoration of its expression resensitized cells to cisplatin by downregulating *cyclin E1 (CCNE1*), a key driver of cell cycle progression (71). miR-34a, previously discussed in prostate cancer, targets CD44 and suppresses angiogenesis and invasion in bladder cancer. A phase I pan-tumor trial with MRX34, a miR-34a mimic, showed early promise but was terminated due to immune-related adverse events. In a separate preclinical model, multicomponent nanoparticles co-delivering siRNA against PD-L1 and miR-34a demonstrated significant antitumor activity in patient-derived xenograft (PDX) models of bladder cancer (72).

#### 4.1.3 Kidney cancer

##### 4.1.3.1 ASO

MG98, an ASO targeting *DNA methyltransferase 1 (DNMT1)*, a key enzyme that plays a fundamental role in regulating gene expression by maintaining DNA methylation. The reduction of *DNMT1* leads to DNA hypomethylation, which can reactivate silenced genes, including tumor suppressor genes. This molecule was conducted until phase II trial in metastatic renal cell carcinoma, but showed no objective responses, questioning the interest if its use in a combination strategy. However, despite being manageable, safety concerns including cytolysis led to the discontinuation of its development (73, 74).

##### 4.1.3.2 siRNA

ARO-HIF2 is a small interfering RNA targeting *hypoxia-inducible factor 2α (HIF-2α)*, a critical driver of clear cell renal cell carcinogenesis due to inactivation of the *VHL* gene. A phase I trial evaluated ARO-HIF2 in 26 patients. Although the drug successfully suppressed *HIF2α* expression in plasma, the objective response rate was limited to 7.7%. Development was halted due to off-target neurotoxicity, but the trial serves as proof-of-concept for siRNA therapies in renal cancer (75, 76).

##### 4.1.3.3 miRNA

Most miRNA studies in kidney cancer remain at the preclinical stage. Notable examples (77, 78) include:

miR-203, which functions as a tumor suppressor by targeting *FGF2*, a growth factor involved in angiogenesis and proliferation, and miR-30a-5p, which inhibits renal cancer cell proliferation by downregulating the *PTEN/AKT* pathway. A summary of all these molecules is provided in **Table 2**.

**Table 2:** Gene silencing therapies in Genitourinary (GU) cancers.

Modality	Drug/Name	Target	Cancer type	Development stage	Reference
ASO	AR-ASO	Androgen Receptor (AR)	Prostate (CRPC)	Preclinical (PDX)	(49)
ASO	Apatorsen (OGX-427)	Hsp27	Prostate, Bladder	Phase I–II	(46, 47)
ASO	Anti-DDX5 ASO	DDX5	Prostate (CRPC)	Preclinical (PDX)	(48)
ASO	Livin-ASO	Livin (IAP family)	Bladder	Preclinical (*in vitro*/*in vivo*)	(67)
ASO	MG98	DNMT1	Renal Cell Carcinoma	Phase II (discontinued)	(73, 74)
miRNA	Anti-miR-221/222	miR-221/222	Prostate	Preclinical (*in vivo*)	(59)
miRNA	miR-34a (DUPA/Nigericin)	CD44	Prostate, Bladder	Preclinical (PDX)	(61)
miRNA	MRX34	miR-34a mimic	Bladder (pan-tumor trial)	Phase I (terminated)	(72)
miRNA	LNP-miR-145	miR-145	Bladder	Preclinical (*in vivo*)	(70)
miRNA	miR-424-5p	Cyclin E1 (CCNE1)	Bladder (cisplatin-R)	Preclinical (*in vitro*)	(71)
miRNA	miR-203	FGF2	Renal Cell Carcinoma	Preclinical	(77)
miRNA	miR-30a-5p	PTN/AKT pathway	Renal Cell Carcinoma	Preclinical	(78)
siRNA	SXL01 (PROSTIRNA)	AR	Prostate	Phase I (terminated)	(50)
siRNA	Epharna	EphA2	Prostate, Bladder, etc.	Phase I	(51)
siRNA	siPARP1	PARP1	Prostate	Preclinical	(52)
siRNA	siClusterin (LNP)	Clusterin	Prostate (CRPC)	Preclinical	(53)
siRNA	siTRIM24 (PSMA-ab)	TRIM24	Prostate (CRPC)	Preclinical	(54)
siRNA	RAG-01	p21 (saRNA)	NMIBC (BCG-unresponsive)	Phase I ongoing	(NCT06351904)
siRNA	siPLK1	PLK1	Bladder	Preclinical (*in vivo*)	(68)
siRNA	siSnail-1	Snail-1	Bladder	Preclinical (*in vitro*)	(69)
siRNA	ARO-HIF2	HIF-2α	Clear Cell RCC	Phase I (discontinued)	(75, 76)

### 4.2 Gastrointestinal tumors

#### 4.2.1 Colorectal cancer (colon and rectum)

Among different target explored to support ASO development, eIF4E is a translation initiation factor overexpressed in approximately 30% of colorectal cancers (79). eIF4E plays a central role in protein synthesis, especially of oncogenic and pro-survival proteins (80). ISIS 183750 is an antisense oligonucleotide that reduces eIF4E mRNA and protein expression. The molecule was developed until phase I/II clinical trial combining ISIS 183750 with irinotecan in metastatic irinotecan-refractory colorectal cancer patients demonstrated good tolerability and disease stabilization (median 22.1 weeks) in 7 out of 15 patients (81). Given irinotecan’s central role in colorectal cancer therapy, the ability of ISIS 183750 to potentially resensitize tumors to this agent represents a highly promising avenue for future therapeutic development.

In cancer, metabolic pathways are profoundly altered, and the glutathione pathway, which includes glutathione S-transferase Pi (GSTP), is often upregulated to counteract oxidative stress and chemotherapy-induced damage (82). GSTP not only detoxifies reactive metabolites but also transmits anti-apoptotic signals by interacting with key regulators of apoptosis, thereby promoting tumor cell survival. Its overexpression has been observed in a wide range of malignancies and is consistently associated with poor treatment response and chemoresistance (83). Targeting GSTP with specific inhibitors or antisense oligonucleotides therefore represents a promising strategy to sensitize cancer cells to chemotherapy and restore apoptotic pathways, highlighting its potential as a therapeutic target. NBF-006 is a novel siRNA targeting *GSTP*. By silencing *GSTP*, NBF-006 aims to limit its anti-apoptotic effect and restore chemosensitivity. In a first-in-human dose-escalation study including CRC patients, NBF-006 was well tolerated with mainly mild side effects and no dose-limiting toxicities. Among CRC patients, one achieved stable disease for 24 weeks, indicating preliminary antitumor activity. These results support further clinical evaluation of NBF-006 in CRC (84).

#### 4.2.2 Pancreatic cancer

*KRAS G12D* is a very common mutation found in pancreatic adenocarcinomas (PDAC) and a key target gene found in various cancer. Mutation confers an active KRAS-GTP protein activating the signaling pathway. KRAS protein as long been considered as “untargetable” due to its 3D conformation conferring an inaccessible binding site of pharmaceutics (85). Different KRAS inhibitors have been developed or are in development, with specific mutation activity (*KRAS G12C, KRAS G12D, KRAS G12V*) (86–88). Different inhibitory strategies are currently under investigation, including inhibition at a common site of KRAS protein (pan-KRAS inhibitors) (89), or molecular glues—an emerging approach that promotes the binding of KRAS to a degradation enzyme (90). But *KRAS* is also the target of a silencing-based approach by siRNA evaluated in phase I studies for pancreatic cancer, in combination with gemcitabine. The siRNA is encapsulated in a biodegradable implant (LODER) placed directly into the tumor and demonstrates encouraging results with no tumor progression in a heavily pretreated cohort. Phase II is ongoing (NCT01188785) (91).

Silencing approaches targeting the tumor microenvironment are also being explored. Atu027, a liposomal siRNA targeting PKN3, a protein that plays a major role in endothelial cell migration during tumor-induced neoangiogenesis. It was evaluated in a Phase I clinical trial (NCT01808638) in combination with gemcitabine for patients with advanced pancreatic cancer. The treatment was well tolerated, with primarily grade 3 laboratory abnormalities and few grade 4 events. In metastatic patients, twice-weekly administration of Atu027 significantly improved progression-free survival (1.6 vs. 2.9 months, p = 0.025) and disease control, supporting the therapeutic potential of endothelial-targeted siRNA strategies (92). Although the absolute magnitude of benefit was modest, these results are encouraging given the poor prognosis of PDAC and the limited availability of effective therapeutic options.

#### 4.2.3 Gastric cancer

There is only preclinical evidence of gene expression silencing therapy in gastric cancer. The most relevant of them are reviewed there. PCDHA11 ASOs targeting protocadherin alpha 11 (*PCDHA11*), a gene promoting gastric cancer cell proliferation and metastasis were also developed. In a high-throughput ASO screening approach, a recent study screened 54 AmNA-modified ASOs for cytological and molecular effets on different tumoral cells. The study selected best candidates that effectively knocked down *PCDHA11* expression. In mouse models of gastric and pancreatic cancer metastasis and subcutaneous tumors, systemic ASO administration inhibited tumor progression. Toxicity was manageable and reversible, and Pcdha11 knockout mice showed normal physiological functions, supporting the safety and therapeutic potential of anti-*PCDHA11* ASOs for gastric and other solid cancers (93). Among siRNA development, two molecules, able to resensitize gastric cancer cells to chemotherapy stood out in our analysis. Long-non coding RNA (lncRNA) *PVT1* has a high expression in GC cells and promotes drug resistance as well as progression. A siRNA mediated lncRNA *PVT1* silencing combined to paclitaxel was able to restore sensitivity to paclitaxel inducing apoptosis, and reducing migratory capability (94). On the other hand, *EZH2* siRNA was able to resensitizing cisplatin-resistant GC to cisplatin since *EZH2* was upregulated in cisplatin-resistant *in vitro* models (95). A summary of all these molecules is provided in **Table 3**.

**Table 3:** Gene silencing therapies in Gastrointestinal (GI) cancers.

Modality	Drug/Name	Target	Cancer type	Development stage	Reference
ASO	ISIS 183750	eIF4E	Colorectal	Phase I/II	(81)
ASO	Anti-PCDHA11 ASO	PCDHA11	Gastric	Preclinical (*in vivo*)	(93)
siRNA	NBF-006	GSTP	Colorectal	Phase I	(84)
siRNA	siPVT1	lncRNA PVT1	Gastric	Preclinical (*in vitro*)	(94)
siRNA	siEZH2	EZH2	Gastric	Preclinical (*in vitro*)	(95)
siRNA	siKRAS G12D (LODER)	KRAS G12D	Pancreatic	Phase I/Phase II ongoing	(91)
siRNA	Atu027	PKN3	Pancreatic	Phase I/II	(92)

## 5 Conclusion

Gene silencing therapies represent a highly promising approach in precision oncology, with the theoretical capacity to target virtually any gene implicated in tumorigenesis. Antisense oligonucleotides, small interfering RNAs, and microRNAs offer a level of specificity rarely achievable with conventional therapies, and they can potentially modulate previously “undruggable” targets. Numerous preclinical models have demonstrated robust efficacy, and the rationale for these approaches is scientifically sound. Gene expression silencing techniques, developed since the 1990s–2000s, belong to the broad family of targeted therapies. They are structured around four major challenges: targeted delivery, molecular specificity, tolerability, and the pharmacological optimization of compounds to ensure their stability within biological tissues. If gene expression silencing techniques tend to progress more slowly than monoclonal antibodies (mAbs), ASOs and siRNAs have been rapidly catching up since 2015–2020, notably due to advances in delivery systems and stabilizing chemical modifications. Despite these advances, gene expression silencing will need to overcome several remaining challenges to reach its full therapeutic potential. Unlike therapies that directly target proteins, such as monoclonal antibodies, gene expression silencing approaches act solely at the mRNA level, without control over post-transcriptional processes that can influence the expression, structure, or final activity of the protein. Gene expression silencing must also address challenges common to all targeted therapies, such as tumor heterogeneity and the emergence of molecular resistance mechanisms. Emerging oligonucleotide-based strategies, including aptamers, which can directly inhibit oncogenic proteins or serve as targeted delivery vehicles, and CRISPR-guided oligonucleotides, which enable precise genome editing, hold promise for gastrointestinal and genitourinary cancers, although their clinical translation remains at an early stage.
